# A Practical Treatise on Disease in Children

**Published:** 1885-01

**Authors:** Robert H. Babcock


### BOOK REVIEWS.

A PRACTICAL TREATISE ON DISEASE IN CHILDREN. *By* EUSTACE SMITH, M. D., F. R. C. P., *Physician to His Majesty the King of the Belgians, Physician to the East London Children's Hospital, and to the Victoria Park Hospital for Diseases of the Chest. Large 8vo., pp. 884. New York:* WILLIAM WOOD & Co., *Publishers.*

Truly in the words of Solomon: "Of the making of many books there is no end." This is particularly pertinent of medical books. Practitioners and students of medicine are actually overwhelmed with announcements of new works, each of which is recommended as the best of its kind. Yet, if one be unwary enough to invest in one of these highly lauded works, he will find, in the majority of cases, that it presents nothing new, nothing original, and is in no respect superior to some other work that has made a valuable part of his library for years; and it ends with his relegating this latest addition to the shelves of his book-case, there to remain, while the old standby is thumbed with ever-increasing respect and fondness. We sincerely deplore this redundancy of medical books of no really superior merits. Our pleasure is therefore the keener when we chance upon a work, of the substantiability and worth of this, whose title heads this notice. That this treatise was greatly needed, when we already possessed works upon diseases of children of such solidity as those of Meigs and Pepper, of Philadelphia, and J. Lewis Smith, of New York, is an open question, but that this contribution of an English practitioner of wide experience, now that it has once appeared among us, will find many readers, give great satisfaction and be laid down only to be frequently reconsulted, is an assertion which will find ready confirmation, we think, in the experience of its possessors. The binding, paper and print are good, and we are especially glad to note an unusual absence of typographical errors. Those we observed are, for the most part, such as will not confuse the reader. On page 92 occurs the statement: "The heart, although itself showing no signs of disease, may have its right ventricle filled with a colorless ante-mortem clot which extends into the ventricle." It is evident that the word "ventricle" which ends the sentence should read auricle. On page 544 mention is made of "Dr. Samson," which doubtless is a transposition of the well known name of Dr. Sanson. The author's style is clear, forcible and for the most part free from tautology. He repeats himself occasionally in enumerating symptoms, but the impression left upon the reader is of a concise handling of the subject without, however, sacrificing such details as render a work complete and interesting. As stated in the preface, the aim of the treatise is to present disease as manifested in children, in a practical manner, and to embody whatever useful hints the author has derived from extended experience in the management of patients of tender years. In this Dr. Smith has been successful. His treatment of children consists mainly in a wise regulation of diet and hygiene, with the administration of as few drugs as possible. When medicinal remedies are resorted to, they are employed upon the same principles as govern their uses in adults. No special chapter is devoted to the consideration of diet, clothing, etc., of infants and young children, but these matters are discussed in the treatment of the individual diseases with a view to whatever modifications of the same may be advisable. He is uncompromising in his antagonism to farinaceous and saccharine articles of food except in very moderate amounts. He considers syrups so deleterious by reason of their likelihood to ferment in the child's stomach, that he employs glycerine instead. He is very partial to the cold douche as a stimulant measure. His therapy is eminently rational, and he does not appear to ride any particular hobby. We cannot point to any line of medicinal treatment that is distinctive or original, yet this department of the work abounds with practical suggestions. The author has taken special pains with the diagnosis of disease, and the reader will find this part clear and to the point. Symptoms are detailed faithfully and understandingly, without such a wearisome mass of unimportant *minutiæ* as some clinical tenchers appear to delight in. The author also expresses himself with much caution and scientific discrimination in dealing with the causation of diseases where definite etiological factors have not been demonstrated. Thus, while admitting chorea to be of influence in the etiology of many cases of endocarditis, he, nevertheless, does not go as far as some of his countrymen, but ranks rheumatism and scarlatina as a much more freqnent cause of heart troubles. What is said upon the morbid anatomy of the various diseases is excellent as far as it goes, but we think the author has made an error in considering this portion of his subject quite so briefly. It might have been rather more extended without any risk of wearying the reader already conversant with the subject. We note that he sides with those pathologists who regard membranous croup as diphtheritic laryngitis. He says, page 95: "In my own experience I can not recall to mind a single case of membranous laryngitis in which some evidence of false membrane in other parts was not obtained. In most cases there was also exudation in the fauces. In a few the membrane had spread down the trachea and the fauces were free; but even in these cases patches of exudation were usually found on examination after death, at the back of the nares."

Although many statements are made with a positiveness born of experience, the work is free from offensive dogmatism, and recognition of the opinions of others is shown liberally. The author's intimate acquaintance with the literature of the subject is unmistakable. We think we speak advisably when we make the statement that this work will rank among the classical productions of the English language in this department, and we heartily recommend it to the careful perusal of practitioners of medicine generally.

ROBERT H. BABCOCK, M. D.

